# The *Arabidopsis* At1g30680 gene encodes a homologue to the phage T7 gp4 protein that has both DNA primase and DNA helicase activities

**DOI:** 10.1186/1471-2229-13-36

**Published:** 2013-03-04

**Authors:** Joann Diray-Arce, Bin Liu, John D Cupp, Travis Hunt, Brent L Nielsen

**Affiliations:** 1 Dept. of Microbiology & Molecular Biology, 775 WIDB, Brigham Young University, Provo, Utah, 84602, USA

**Keywords:** DNA primase, DNA helicase, bacteriophage T7 gp4, Twinkle, Organelle DNA replication

## Abstract

**Background:**

The *Arabidopsis thaliana* genome encodes a homologue of the full-length bacteriophage T7 gp4 protein, which is also homologous to the eukaryotic Twinkle protein. While the phage protein has both DNA primase and DNA helicase activities, in animal cells Twinkle is localized to mitochondria and has only DNA helicase activity due to sequence changes in the DNA primase domain. However, *Arabidopsis* and other plant Twinkle homologues retain sequence homology for both functional domains of the phage protein. The *Arabidopsis* Twinkle homologue has been shown by others to be dual targeted to mitochondria and chloroplasts.

**Results:**

To determine the functional activity of the *Arabidopsis* protein we obtained the gene for the full-length *Arabidopsis* protein and expressed it in bacteria. The purified protein was shown to have both DNA primase and DNA helicase activities. Western blot and qRT-PCR analysis indicated that the *Arabidopsis* gene is expressed most abundantly in young leaves and shoot apex tissue, as expected if this protein plays a role in organelle DNA replication. This expression is closely correlated with the expression of organelle-localized DNA polymerase in the same tissues. Homologues from other plant species show close similarity by phylogenetic analysis.

**Conclusions:**

The results presented here indicate that the *Arabidopsis* phage T7 gp4/Twinkle homologue has both DNA primase and DNA helicase activities and may provide these functions for organelle DNA replication.

## Background

DNA replication involves the coordinated activity of several enzymes and proteins. These enzymes assist with the unwinding, separation, and copying of double stranded DNA to produce new identical DNA copies [1]. DNA helicase translocates unidirectionally along one strand of the nucleic acid to facilitate replication initiation. The helicase utilizes ATP hydrolysis to separate the DNA double helix into individual strands [2,3]. DNA primase catalyzes the formation of short RNA oligonucleotides used as primers to begin DNA synthesis [4]. DNA polymerase uses the primers and extends the 3' end of the nucleotide chain by adding nucleotides matched to the template strand [1].

Individual genes are usually responsible for encoding each replication enzyme activity. However, bacteriophage T7 gene 4 protein (T7 gp4) and similar proteins from T3, P4 and other phages [4] encode a single protein with both DNA helicase and DNA primase domains. T7 phage has two forms of gp4 protein that are both required for phage genome replication. The longer form has two zinc motifs and has both DNA primase and helicase activity while the shorter one retains only DNA helicase activity [5].

Most eukaryotic organisms have a homologue of the T7 gp4 protein that has been named Twinkle (T7 gp4-like protein with intramitochondrial nucleoid localization). This protein shares close sequence similarity with the bacteriophage T7 gp4 primase-helicase protein [6,7]. Twinkle is a hexameric DNA helicase at the mitochondrial DNA replication fork which unwinds sections of double-stranded DNA [8,9]. The Twinkle homologue lacks DNA primase activity in higher eukaryotes but is suggested to have this activity in *Plasmodium* species [6,10] and *Arabidopsis thaliana* and other plants [11,12]. This protein is assumed to play a key role in mitochondrial DNA (mtDNA) replication as it localizes in the mitochondrial nucleoid and matrix. In maize, Twinkle has also been found associated with the chloroplast nucleoid [13], suggesting that this protein may function in both mitochondria and chloroplasts.

Mutations in Twinkle result in mitochondrial-associated diseases in humans [6,14] and mice [15,16]. In humans, coding region mutations in this gene have been linked with autosomal dominant progressive external ophthalmoplegia (adPEO) and are often associated with multiple mtDNA deletions, suggesting a role in mtDNA replication [6]. In mice, Twinkle expression reduction by RNAi resulted in a rapid drop in mtDNA copy number [6,17] while overexpression of the protein led to increases in mtDNA copy number in muscle and heart tissue [15,18].

When the amino acid sequences of Twinkle homologues from a wide variety of eukaryotic species are compared, high homology in the conserved Walker motifs for the DNA helicase domain of the protein has been observed, as summarized in two review papers [4,5]. Critical differences were observed in the primase domain of Twinkle in some model organisms when compared to the N-terminal end of the T7 gp4 protein [19]. The location of the (nonfunctional) primase domain in human Twinkle is at the N-terminal portion of the protein, the same as in phage T7 gp4 and in DNAG-like primases in bacteria and phage [4,11]. But unlike T7 gp4, the N-terminal domain of human Twinkle lacks several motifs required for primer synthesis in T7 gp4, thus leading to the prediction that the Twinkle N-terminal region is generally inactive in humans and metazoa in general [5]. The T7 gp4 protein contains a beta sheet structure and cysteine residues forming two zinc fingers [7] in Motif 1. The N-terminal end of the primase domain of T7 gp4 contains a zinc finger motif but Twinkle in most metazoan species lacks the zinc-binding domain necessary for DNA and amino acid binding for polymerization of the primer [5]. Also, human Twinkle does not contain the conserved cysteine residues of the zinc-finger motif critical for DNA binding and primase activity [20]. The zinc finger motif in the primase domain synthesizes pppAC oligonucleotide primers important for the initial step of sequence-specific primer synthesis at the sequence 5^′^-GTC-3^′^[21]. The Twinkle protein from *Arabidopsis thaliana* contains the conserved sequence elements and is predicted to have both DNA primase and DNA helicase activities.

The *Arabidopsis* genome contains two homologues of the bacteriophage T7 gp4 protein. The first (At1g30680) shares homology with the conserved motifs of the DNA primase and DNA helicase domains [5]. The coding sequence predicts a protein of about 80 kDa, which is larger than the full-length 63,000 kDa T7 gp4 protein but similar to the sizes of Twinkle homologues reported in eukaryotes. The second *Arabidopsis* homologue is truncated, sharing the N-terminal primase domain but entirely lacking the C-terminal helicase domain, with a predicted size of ~38 kDa (At1g30660). The truncated gene will be designated as a primase homologue, while the full-length gene will be designated as a Twinkle homologue in this paper.

We show here that the *Arabidopsis* T7 gp4 homologue has both DNA primase and DNA helicase activities, the first such report from a higher eukaryote. The gene for this protein is highly expressed in rapidly growing plant tissues and is correlated with organelle DNA polymerase gene expression.

## Results

### Expression of the *Arabidopsis* protein in *E. coli* and demonstration of DNA primase activity

**Figure 1:**
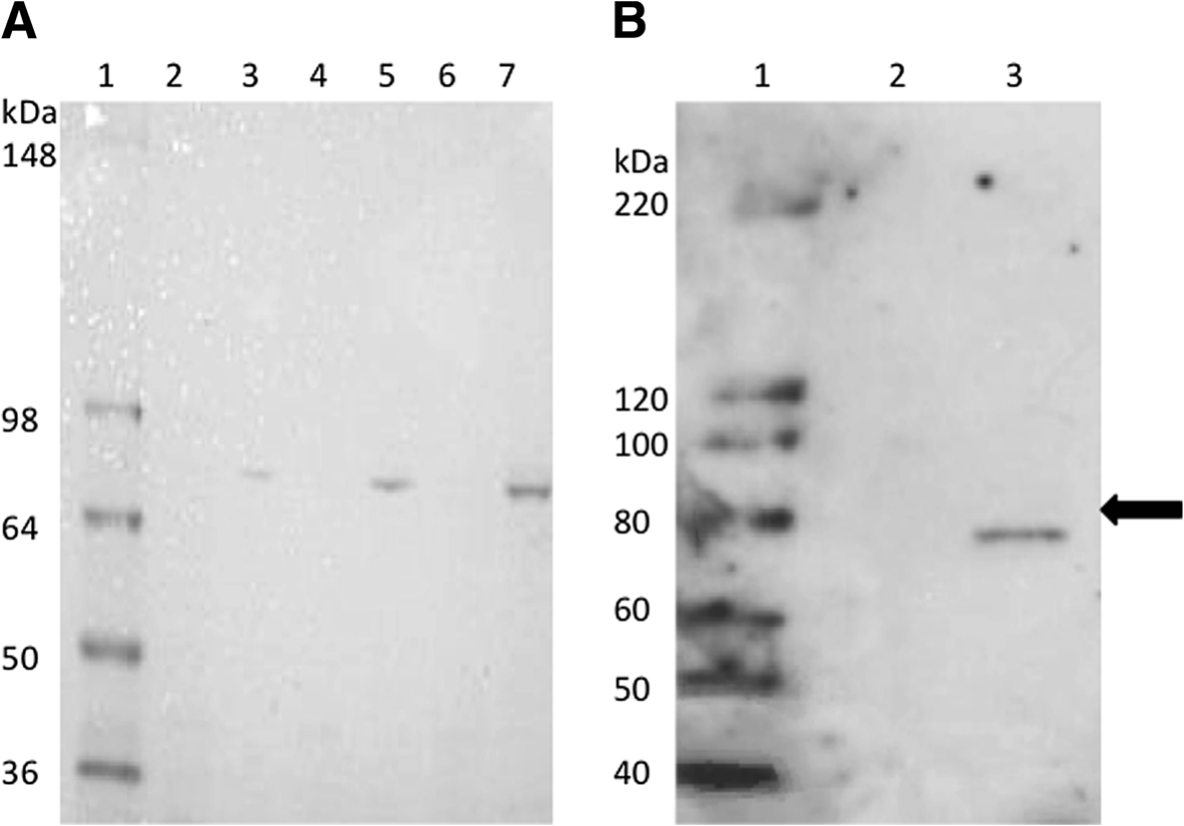
**Purification of the recombinant protein.** Panel **A** shows the Coomassie Blue-stained gel, with increasing amounts of the purified recombinant (lanes 3, 5 and 7) and control (lanes 2, 4 and 6) protein, from left to right. Lane 1, protein molecular weight markers (Invitrogen SeeBlue 2 markers). Lanes 2 and 3, 0.195 ng; lanes 4 and 5, 0.39 ng; lanes 6 and 7, 0.585 ng. Panel **B** shows a western blot of the purified protein using antibody against the *Arabidopsis* Twinkle homologue. Lane 1 contains molecular weight markers (Invitrogen Magic Markers). Lane 2, control protein; lane 3, 0.5 ng purified recombinant protein. The arrow at the right indicates 80 kDa, the length of the full-length *Arabidopsis* gene product. The recombinant protein is slightly smaller (~74 kDa) as it lacks the N-terminal localization sequence.

**Figure 2:**
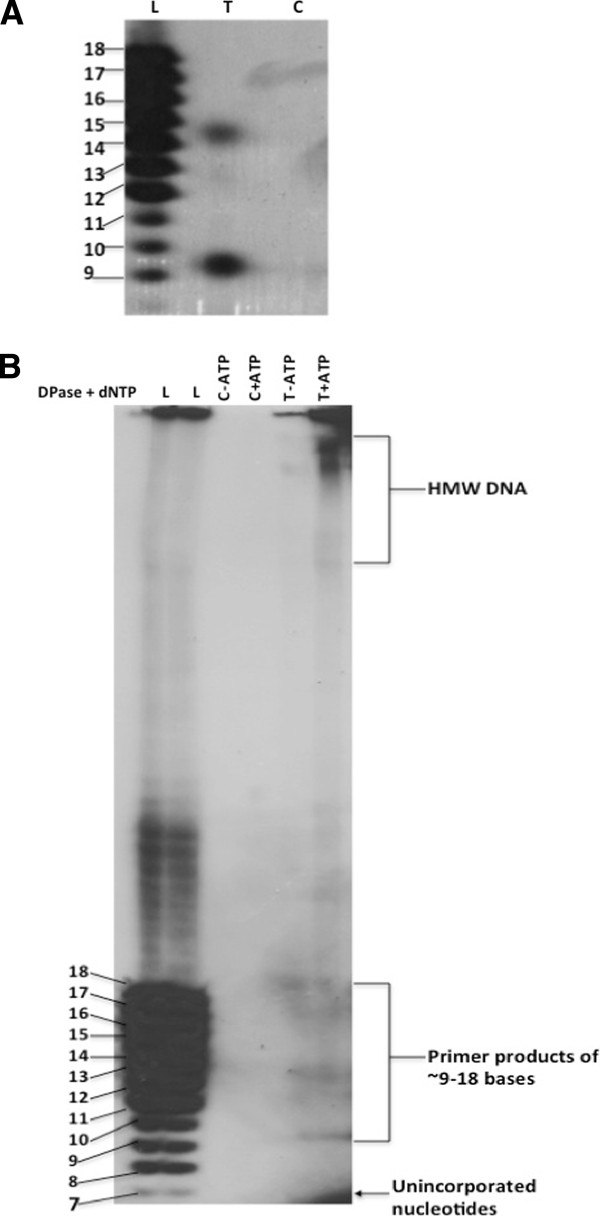
**DNA primase assay.** The recombinant Twinkle homologue purified from *E. coli* cells was tested for DNA primase activity. Panel **A**, lane L (DNA single-base ladder), oligo dT_9-18_ included as size markers (same for panel B). Lane T, reaction products with the recombinant protein. Lane C, reaction products using a bacterial fraction with the empty vector as control. Panel **B** shows incorporation of primers into high molecular weight DNA in the presence (lane 6) but not the absence (lane 5) of *E. coli* DNA polymerase I and dNTPs. Lanes 3 and 4 are the control protein fraction in the absence (lane 3) and presence (lane 4) of DNA polymerase I and dNTPs.

The full-length cDNA for the *Arabidopsis* Twinkle gene was obtained and cloned into a bacterial expression vector to produce protein for enzymatic activity assays. The purified protein showed a predominant band of the proper size by gel staining (Figure 1A). Its identity as the expressed protein was confirmed by western blot analysis using an antibody against a synthetic peptide from the *Arabidopsis* protein sequence (Figure 1B). The recombinant protein product is smaller (~74 kDa) than the full-length coding region of the Twinkle homologue since it lacks the N-terminal organelle targeting sequence. The purified protein was used for an *in vitro* assay for DNA primase activity. Gel analysis of the reaction products indicates that the protein is capable of producing RNA primers of ~ 9–18 bases from a single-stranded DNA template (Figure 2). Stronger intensity of the primers of 9 and 14 bases was consistently observed (close-up shown in Figure 2A), and are similar in size to products reported for other DNA primases [22]. The primers were capable of being extended by DNA polymerase into high molecular weight DNA (Figure 2B), which is a fundamental property of a DNA primase that generates primers for DNA replication. The primer bands are absent in the control lanes (protein from bacteria with the empty vector lacking the *Arabidopsis* gene), indicating that this activity is not due to bacterial DNA primase contamination of the purified recombinant protein. This provides clear evidence for the function of the *Arabidopsis* Twinkle homologue as an active DNA primase, the first such report in a higher eukaryote.

### DNA helicase activity of the *Arabidopsis* Twinkle homologue protein

**Figure 3:**
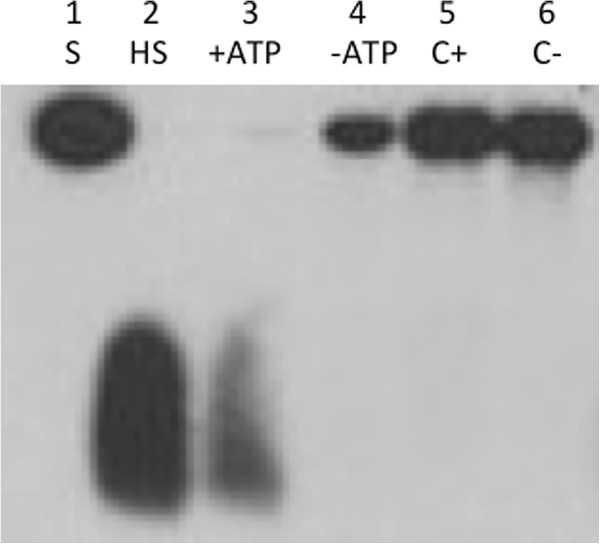
**DNA helicase assay.** The recombinant Twinkle homologue purified from *E. coli* cells was tested for DNA helicase activity as described in the text. Lane 1 is the control substrate (S). Lane 2 is the heated control (HS), showing separation of the short labeled oligo from the substrate, which runs in this gel as a leading band with a diffuse smear; lane 3 (T+ATP), reaction using the purified recombinant protein with ATP; lane 4 (T-ATP), same reaction without ATP, lane 5 (C+ATP), control protein from *E. coli* cells lacking the expression construct with ATP, lane 6 (C–ATP), same reaction but without ATP.

The purified recombinant protein was also assayed for DNA helicase activity. The results indicate that the protein indeed has ATP-dependent DNA helicase activity as predicted (Figure 3). The control protein preparation (vector with no insert) lacked DNA helicase activity in the presence or absence of ATP (Figure 3 lanes 5 and 6). The activity is similar to the DNA unwinding activity we previously detected in soybean mitochondrial extracts [23]. The results from the biochemical assays indicate that the *Arabidopsis* Twinkle homologue has both DNA primase and helicase activities, similar to the phage T7 gp4 protein.

### Western blot analysis of *Arabidopsis* Twinkle homologue expression

**Figure 4:**
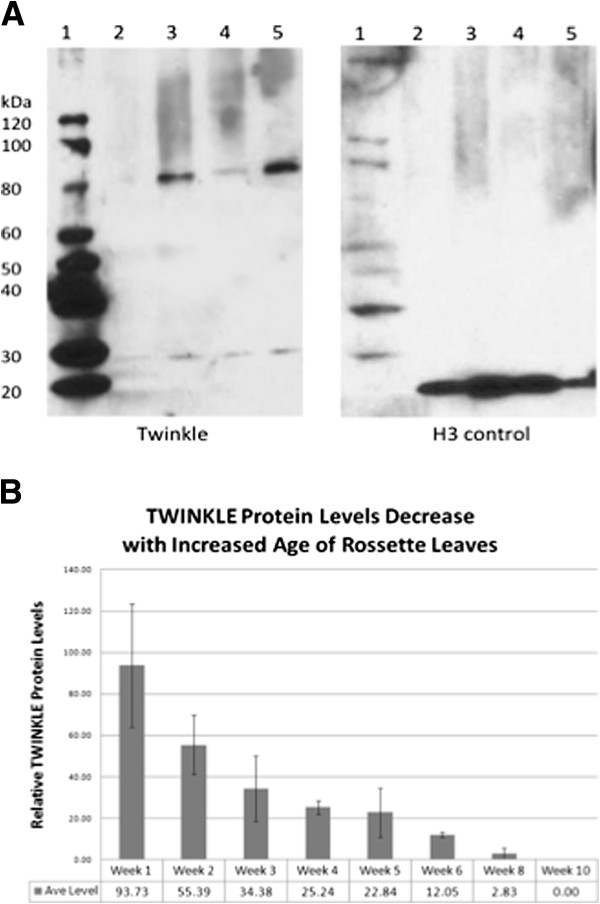
**Western blot analysis of *Arabidopsis *Twinkle homologue expression. A**. Lane 1, Molecular weight markers, Lane 2, leaf tissue from 6-week plants; lane 3, shoot apex tissue; lane 4, total plant tissue protein; lane 5, cotyledon protein. The panel on the left was incubated with antibody against the Twinkle protein. The panel on the right was incubated with histone H3 antibody as a loading control. **B**. Relative levels of Twinkle protein relative to a nuclear tubulin protein control in *Arabidopsis* seedlings harvested at the times indicated. The average of three independent western blots is shown for each time point (weeks 1–5 and 10). Error bars indicate the SEM (standard error of the mean).

Western blot analysis of Twinkle protein expression levels in different *Arabidopsis* tissues shows that the protein is most abundant in meristem and young leaf tissue and nearly undetectable in mature leaves (Figure 4A). Total rosette leaf tissue from plants was collected at weekly intervals and total protein was recovered from each sample for western blot analysis. The results show relatively high levels of the Twinkle protein in weeks 1–3 of growth, with a subsequent rapid drop in levels until the protein is nearly undetectable after week 5 (Figure 4B). These results are compatible with those reported from the different tissues (Figure 4A). Western blot analysis indicated the presence of Twinkle in isolated mitochondria and chloroplasts of *Arabidopsis* (data not shown).

### Analysis of *Arabidopsis* Twinkle homologue expression in different tissues by qRT-PCR

**Figure 5:**
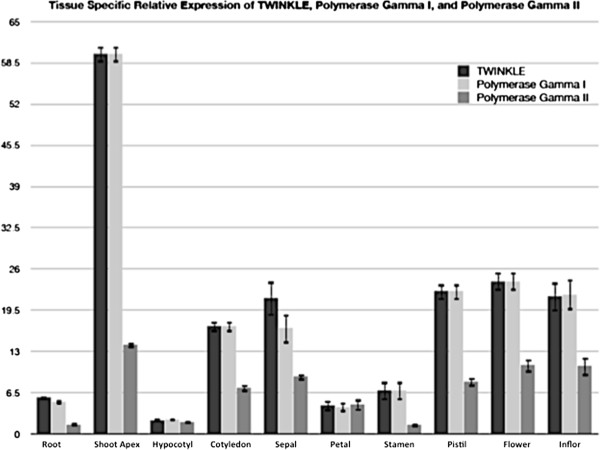
**RT-qPCR analysis of the *Arabidopsis *Twinkle homologue gene expression relative to organellar localized DNA polymerases in various tissues.** The relative abundance of Twinkle and the two organellar DNA polymerases (Polymerase gamma I and Polymerase gamma II) is shown. Expression varied among selected organs with highest expression in the shoot apex. The relative expression of Twinkle follows the expression levels of DNA polymerase gamma I. Error bars indicate SEM of three replicates. The Y axis indicates relative expression (log_2_) normalized to nuclear actin gene expression. Inflor, inflorescence.

Quantitative reverse transcriptase PCR analysis of cDNA generated from different tissues indicate that the *Arabidopsis* Twinkle gene is expressed at the highest level in the shoot apex (Figure 5), as expected if the Twinkle protein plays a role in organelle DNA replication in rapidly growing tissues. Twinkle is also expressed at relatively high levels in other developing tissues, especially cotyledons and different parts of flowers including sepals, pistils and the inflorescence (Figure 5). Interestingly, expression levels of Twinkle are very similar to expression levels of DNA Pol gamma I (Figure 5), a dual-targeted DNA polymerase that has been shown to play a role in plant organelle DNA replication and repair [24]. The expression of DNA Pol gamma II is also generally highest in the same tissues that have high Twinkle expression (Figure 5). The similar levels of expression of Twinkle and the organelle-localized DNA polymerases [25] suggest that Twinkle may play a role in replication of organelle DNA.

### Analysis of Twinkle DNA and protein sequences

Two separate research groups have reported on the comparison of the amino acid sequences of Twinkle homologues from a wide variety of eukaryotic species, and have shown that there is high homology in the conserved Walker motifs for the C-terminal DNA helicase domain of the *Arabidopsis* protein [4,5]. The human, *Drosophila* and *C. elegans* Twinkle homologues have DNA helicase activity but lack DNA primase activity [4,5]. Upon close examination of the amino acid sequence encoding the primase domain at the N-terminal end of the protein in the plant and animal proteins, some critical differences are apparent. Two zinc fingers formed by cysteine residues in Motif 1 are present in the T7 gp4 protein [7] and in homologues from most eukaryotes, but the four cysteines that form the zinc fingers are absent in metazoans, including humans [4,5]. Analysis of the amino acid sequence alignment of the Twinkle homologues against the T7 gp4 protein shows that only the *Arabidopsis* and other plant Twinkle homologues share all highly conserved elements with the T7 gp4 protein [5]. Additional important differences are observed in other conserved motifs within the primase region of the protein in humans, *Drosophila* and *C. elegans*, while the sequences from a number of lower eukaryotes share the conserved elements with T7 gp4 protein [5]. In particular, the human homologue lacks both zinc finger domains in Motif 1, and the human and *Drosophila* sequences lack the highly conserved residues found in Motif IV and Motif V.

**Figure 6:**
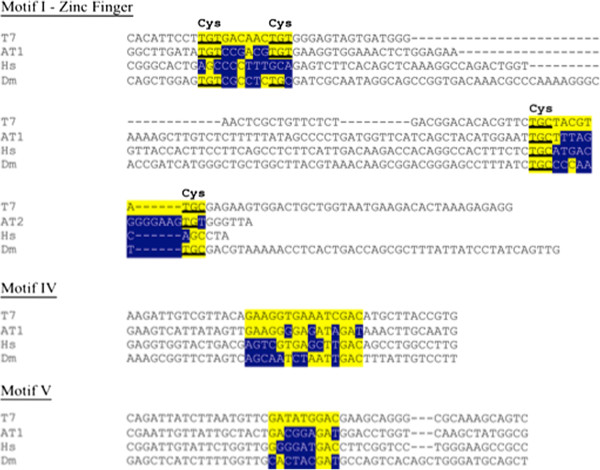
**A. DNA sequence alignments of some Twinkle primase domain conserved regions to show the extent of changes between different organisms.** The DNA sequences for the Twinkle protein from T7, *Arabidopsis* (At1g30680), human (Hs) and *Drosophila* (Dm) are shown for the conserved motifs I, IV and V. The locations of the cysteine residues in Motif I are indicated above the sequence while the corresponding codon sequence is underlined in the DNA sequence. The central conserved elements of each motif are shaded yellow. Base differences from the T7 gp4 sequence are shaded dark blue with white lettering.

The *Arabidopsis thaliana* Twinkle protein contains the conserved sequence elements and is predicted to have both DNA primase and DNA helicase activities [4]. While the previous analysis of the amino acid sequences of these proteins identified critical differences at some conserved sites in the primase domain region of the protein in metazoa, including the absence of the cysteine residues needed to form the zinc fingers [4,5], we wanted to know if these changes were due to minor mutations in the sequence. However, DNA sequence analysis indicates that the differences in amino acid sequence of the homologues in human and *Drosophila* are not due to single base changes but are due to more significant alterations in the DNA sequence (Figure 6). The base sequence differences that are present in the *Arabidopsis* Twinkle primase domain as compared to the T7 gp4 protein mostly occur in the third position of the codons and do not alter the amino acid sequence.

**Figure 7:**
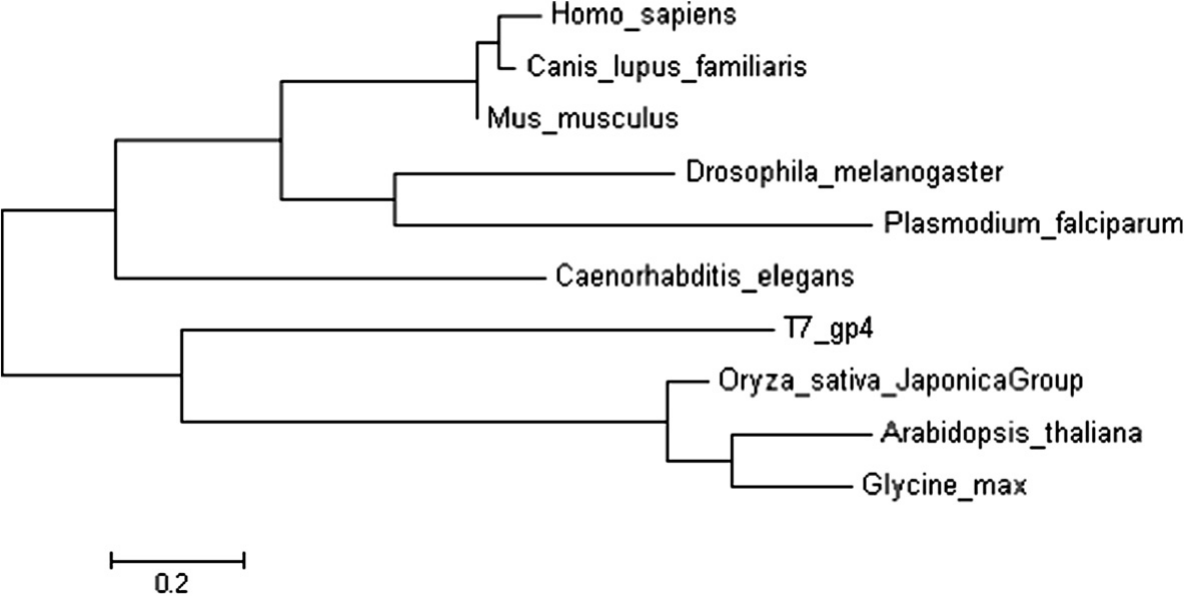
**Phylogenetic analysis of the T7 gp4 protein, plant homologues, and selected eukaryotic Twinkle protein homologues.** Molecular phylogenetic analysis was performed using the maximum likelihood method. The scale bar indicates the number of substitutions per site.

Phylogenetic analysis of amino acid sequences of Twinkle homologues from several plants and other species shows that the *Arabidopsis* and plant homologues are closely clustered and are most similar to the bacteriophage T7 gp4 protein (Figure 7). The relationship between Twinkle proteins is supported by maximum likelihood phylogenetic analysis of taxonomic samples of Twinkle homologues. This suggests that the Twinkle homologues from humans and other animals are most distantly related to the T7 gp4 protein, supporting the observations from direct DNA and amino acid sequence alignments.

## Discussion

Twinkle has been shown to be the replicative DNA helicase in mitochondria of eukaryotic cells, and mutations that abolish expression of this gene are lethal in animal cells [6,14,15,26]. Twinkle is a homologue of the bacteriophage T7 gp4 protein, which has both DNA primase and DNA helicase activities and contains the highly characterized TOPRIM domain that is conserved in DNA primases, topoisomerases and OLD family nucleases [4]. However, until the present work no Twinkle homologue from a higher eukaryote has been shown to have DNA primase activity. Shutt and Gray have analyzed the sequence of Twinkle homologues from several eukaryote species and have proposed that in addition to being the DNA helicase, Twinkle may also serve as the mitochondrial DNA primase in most eukaryotes except metazoa [5]. As far as we know our present report is the first to show that the Twinkle homologue in a plant species (*Arabidopsis*) has both DNA primase and DNA helicase activities. Other than the truncated primase homologue already mentioned (At1g30660; but there is no information available about whether this protein is functional) no other bacterial or phage-type DNA primase homologues have been found in the *Arabidopsis* genome sequence.

Sequence analysis provides an explanation of why the plant homologue has both activities while the animal homologues lack DNA primase activity (Figure 6). The absence of primase activity in human Twinkle is likely due to the lack of the zinc finger motifs formed by 4 cysteine residues near the N-terminal end of the protein, as well as other amino acid sequence differences at conserved sequences in the primase domain of the protein which have been shown to be responsible for the primase activity (Figure 6) [4]. Sequence variation occurs in other metazoan species, and while some have the zinc fingers, they have differences at other conserved motifs. The *Arabidopsis* homologue, in contrast, retains all conserved motifs [4]. Phylogenetic analysis further supports these findings, indicating that the plant Twinkle homologues are most closely related to the T7 gp4 protein, while the animal homologues are quite distantly related. These results suggest that the bifunctional T7 gp4 homologue may be conserved in higher plants.

The *Arabidopsis* Twinkle protein may function both in mitochondria and chloroplasts, as this protein has been shown to be dual-targeted to both organelles [27,28]. These reports are based on the analysis of predicted N-terminal targeting sequences of a number of nuclear-encoded *Arabidopsis* proteins fused with the GFP coding region. However, it has been shown that targeting of fusion proteins can be affected by the context of the N-terminal sequence with the GFP sequence [28,29]. A recent report on the maize plastid proteome has shown the presence of Twinkle in the chloroplast nucleoid [13].

Mitochondrial genomes range widely in size, from about 16.5 kbp in vertebrates and invertebrates, to 70–100 kbp in yeast and 200–2000 kbp in plants. The replication of animal mtDNA has been characterized in great detail, and in the original model each strand of the duplex DNA replicates at a different time, with the initial replication primed by a short transcript synthesized by the mitochondrial RNA polymerase [30]. The second strand replicates only when it becomes single stranded by progression of the first strand, allowing formation of a characteristic structure to facilitate replication initiation of this strand. In yeast and plants, mtDNA replication appears to be more complex, and may involve a recombination-dependent replication mechanism [23,31-34]. In this case DNA priming may not be required if invading strands provide the priming function for DNA synthesis. However, even in phage systems that replicate by a recombination mechanism a DNA primase is still required for priming synthesis at lagging strands during some phases of DNA replication [4].

A distinct mtDNA primase activity has been reported in some animal and protist cells and mtDNA primase activity has been reported in human cells, but no distinct human protein with this activity has yet been identified. It has been suggested that the DNA primase in animal cells is tightly associated with the mtDNA (γ) polymerase, and is thus difficult to isolate separately [35]. In a trypanosome a mtDNA primase of 70 kDa has been reported [36], while in yeast a mtDNA primase of 67 kDa has been characterized [37], which are both close to the size of T7 gp4 and Twinkle. Our understanding of animal mtDNA replication is complicated by reports of strand-coupled bidirectional replication from a single replication origin, which by its nature should require a DNA primase to synthesize primers for the lagging strand [30,38,39]. It is unclear whether a separate mtDNA primase is present or required in species (including human) with highly compact mitochondrial genomes [40]. Recently it has been shown that *in vitro*, human mitochondrial RNA polymerase is responsible for priming lagging strand mtDNA synthesis. It may be possible that priming of replication of the small animal mitochondrial genome is provided by short transcripts synthesized by the mitochondrial RNA polymerase [40,41].

A DNA primase has been purified and characterized from pea chloroplasts [42], and primers synthesized by that preparation are similar in size to primers synthesized by the purified *Arabidopsis* Twinkle homologue. The pea enzyme is larger (~90 kDa) than the *Arabidopsis* Twinkle homologue, but it was not characterized for DNA helicase activity. CtDNA replication involves multiple replication origins and bidirectional DNA synthesis [42,43], which would require DNA primase activity for lagging strand synthesis.

Organelle DNA replication appears to be different in plants (as compared to animals), which have very large and complex mitochondrial genomes and likely require multiple sites of lagging strand DNA synthesis. The role of recombination-mediated replication [33,34] may reduce the need for primase-synthesized primers for organelle DNA replication, as an invading DNA strand could provide the 3^′^ ends for DNA synthesis. However, even in this case it is likely that organelle DNA primase is required in plants. Bacteriophage T4 replicates by multiple mechanisms, including recombination-dependent replication, and requires a DNA primase. The observations that the *Arabidopsis* Twinkle protein is expressed at highest levels in the shoot apex and other developing tissues including young leaves provides strong support for a role of the Twinkle homologue in plant organelle DNA replication, similar to its role in other species [4,5].

Mutations in human Twinkle have been shown to lead to a drastic reduction in mtDNA copy number and disease [17]. RNAi-mediated reduction of Twinkle expression in cultured human cells was found to lead to a rapid drop in mtDNA copy number, while overexpression of Twinkle in mouse tissue was associated with an increase in mtDNA copy number [15,26]. In each of these cases the effect has been associated with the DNA helicase activity of the protein. We showed that this single protein from *Arabidopsis* has both DNA primase and DNA helicase activities in vitro, the same activity as the bacteriophage T7 gp4 protein.

## Conclusion

The *Arabidopsis* homologue of the bacteriophage T7 gp4 protein has been shown to have both DNA primase and DNA helicase activities similar to the phage protein. It is expressed at highest levels in actively growing tissues, suggesting that it could play a role in organelle DNA replication. Two DNA polymerases have been identified in plants, and both have been reported to be dual targeted to mitochondria and chloroplasts [28,44]. It is likely that this *Arabidopsis* phage T7 gp4 homologue functions along with one or both of these DNA polymerases to accomplish organelle DNA replication. Even if the mtDNA replicates by a recombination-dependent mechanism as suggested by some [23,33,34], DNA priming may be required for lagging-strand DNA replication. This *Arabidopsis* protein may also play a role in control of plant mtDNA (and possibly also ctDNA) copy number as observed in animals [5,17], but this determination will require additional experiments, which will be the subject of future work in our lab.

## Methods

### Identification of an *Arabidopsis* Twinkle homologue

A full-length Twinkle homologue was identified in the *Arabidopsis thaliana* genome (At1g30680, protein molecular weight of 80,401.9 Da). A second, truncated homologue is also present (At1g30660, molecular weight of 37,806.9 Da) near the first gene, but contains only the primase domain of the protein and ends near the linker region [45] joining the primase and helicase domains. Only the full-length gene (At1g30680) was examined in this study.

### Recombinant expression of the *Arabidopsis* Twinkle homologue

The full-length cDNA for At1g30680 was obtained from Riken (Japan). The full-length coding region for this gene predicts a polypeptide of 709 amino acids, and the MitoProt program [46] predicts the cleavage site after amino acid 91, which is prior to the conserved elements including the zinc fingers in the DNA primase domain of the protein. We generated a construct of the entire conserved coding region of the gene but lacking the DNA sequence for the N-terminal 91 amino acids in the pEXP5-NT/TOPO expression vector (Invitrogen). The construct was then transformed into the *E. coli* BL21 strain (Invitrogen). A total volume of 500 ml of LB was used to grow the bacteria. After it reached O.D._600_ 0.4-0.6, IPTG was added to the medium to a final concentration of 0.5 mM to induce the expression of the targeted protein. The cells were grown at 30°C for an additional 4 hr and harvested by centrifugation. A control strain containing an empty vector lacking the gene insert was grown under identical conditions. The recombinant protein and control sample were purified under identical conditions using ProBond Nickel-chelating resin (Invitrogen). Native conditions were used and the purification was performed as described in the manual. Protein purity was analyzed by gel electrophoresis and western blot analysis.

### DNA primase activity assay

DNA primase activity of the recombinant protein was detected using a previously published procedure [42] using single-stranded M13 DNA as template. A control bacterial fraction was included to eliminate the possibility that bacterial DNA primase was present in the recombinant protein fraction. Single-stranded M13 DNA was incubated with 0.5 ng of the ProBond-purified recombinant or control protein fraction in the presence of rNTPs including α^32^P-ATP (MP Biomedical). The reaction products were separated in a 20% denaturing polyacrylamide gel (6% urea in 1X TBE). End-labeled oligo(dT)_12–18_ was used as size markers. After electrophoresis the gel was dried and exposed to X-ray film.

### DNA helicase activity assay

DNA helicase activity of the ProBond-purified recombinant protein was assayed according to the procedure of Song [47].The substrate was prepared by annealing (heating for 5 min to 65°C in 40 mM Tris–HCl, pH 7.8, 50 mM NaCl and slowly cooling to room temperature for 20–30 min) single-stranded M13 circular DNA with a complementary oligonucleotide (5^′^ GTAAAACGACGGCCAGT 3^′^) labeled at the 5^′^ end using T4 polynucleotide kinase (New England Biolabs) and γ^32^P-ATP (MP Biomedical). The substrate was incubated with 0.5 ng of the recombinant protein in reaction buffer (10 mM Tris–HCl, pH 8.0, 8 mM MgCl_2_, 1 mM dithiothreitol, 5 mM ATP, 1 ng ^32^P-labeled helicase substrate) for 30 min, after which the reaction was terminated by adding EDTA to 2 mM, and the reaction products were separated by electrophoresis through a native TBE 6% polyacrylamide gel. The same bacterial protein control was included. The gel was then dried and exposed to X-ray film.

### Western blot analysis of Twinkle homologue expression in different tissues

Protein fractions were prepared from different tissues of *Arabidopsis thaliana* by grinding in liquid nitrogen and suspending in 1X SDS-loading buffer. The proteins were heated to 95°C for 5 min and separated by electrophoresis in 8-20% SDS-PAGE gels. Proteins were transferred to PVDF membrane and after blocking in 5% skim milk the membrane was incubated with antibody that had been raised in rabbit (by Sigma-Genosys) against a synthetic peptide from a unique region of the Twinkle protein (KASRIVIATDGDGPG). This sequence is shared in both the full-length and truncated *Arabidopsis* genes (At1g30680 and At1g30660). The sequence of the peptide antigen was compared to the entire *Arabidopsis* proteome to ensure it does not share homology with any other protein besides the Twinkle homologues (NCBI-Blast). A control blot against the histone H3 protein was performed for normalization of signal strength. Bound antibody was detected using the Pierce Supersignal Western Chemiluminescence kit followed by exposure to X-ray film.

For time course analysis, total leaf tissue was extracted from *Arabidopsis* plants at weekly intervals starting at 1 week of age. The tissue was flash frozen in liquid nitrogen and stored at -80°C. Total protein was extracted from 50 mg of crushed and homogenized tissue with 1X SDS-loading buffer [48]. Samples were quantified (BioRad RC DC protein assay kit) and normalized prior to electrophoresis by SDS-PAGE. Western blots were conducted as described above. Protein levels were determined by averaging mean pixel intensities measured with Un-Scan-It software (Silk Scientific, Orem, Utah) from three independent western blots.

### Gene expression analysis by qRT-PCR

RNA was isolated using the PureLink RNA Mini Kit (Invitrogen) from young *Arabidopsis* seedlings. For very small tissues more than 200 young plants were used to obtain enough sample. Shoot apex tissues were taken as the very tip of the young shoots and include the apical meristem. The RNA was quantified and 1 μg was added to a reverse transcription reaction with SuperScriptIII (Invitrogen). The cDNAs from these reactions were diluted with 100 μl of sterile water and added to qPCR reactions as described by the manufacturer (Roche). qPCR reactions consisted of 1X SYBR Green PCR Master Mix (Roche), and 50 nM of each primer. Primers for the *Arabidopsis* Twinkle gene were 5^′^-TCCCCAGAGTCCCAACTCCTGTTGA-3^′^ and 5^′^-TCCCTGTTCCGCCAATTTACGCC-3^′^; for DNA polymerase gamma 1 (At3g20540) were 5^′^-CCTGAATACCGTTCACGTGCCCA-3^′^ and 5^′^-AGCCGCACTTCCCTGAACAGGA-3^′^, and for DNA polymerase gamma 2 (At1g50840) were 5^′^- TTCCGGCGTCAAAGTCACGTGC-3^′^ and 5^′^-TGCACTTCCCTGGACTGGAGTGT-3^′^. Reactions were carried out in a LightCycler 480 System (Roche) for 45 cycles (95°C for 10 sec, 58°C for 10 sec, 72°C for 20 sec) after initial 5 min incubation at 95°C. The fold changes in gene expression were calculated using the ΔΔCt method [49], with the Tub 4 tubulin gene (At5g44340) as an internal control.

### Phylogenetic analysis

Protein sequences for Twinkle homologues were downloaded from Gen Bank with the following accession numbers: *Homo sapiens* (NP_068602.2), *Caenorhabditis elegans* (F46G11.1), *Drosophila melanogaster* (NP_609318.1), *Plasmodium falciparum* (NP_702000.1), T7 gp4 (P03692.1), *Mus musculus* (AAL27647.1), *Canis lupus familiaris* (XP_543974.1), *Arabidopsis thaliana* (ACI49800.1), *Glycine max* (XP_003546288.1), and *Oryza sativa* Japonica group (BAD46002.1). Multiple sequence alignment was performed using MUSCLE [50] and the evolutionary history was inferred by using the Maximum Likelihood method based on the JTT matrix-based model [51]. The tree with the highest log likelihood (−3556.6701) is shown. Initial trees for the heuristic search were obtained automatically as follows. When the number of common sites was < 100 or less than one fourth of the total number of sites, the maximum parsimony method was used; otherwise BIONJ method with MCL distance matrix was used. The tree is drawn to scale, with branch lengths measured as the number of substitutions per site. The analysis involved 10 amino acid-coding sequences. The coding data was translated assuming a standard genetic code table. All positions with less than 95% site coverage were eliminated. That is, fewer than 5% alignment gaps, missing data, and ambiguous bases were allowed at any position. There were a total of 199 positions in the final dataset. Evolutionary analyses were conducted in MEGA 5 [50].

## Abbreviations

qRT-PCR: quantitative reverse-transcriptase PCR; mtDNA: mitochondrial DNA; ctDNA: chloroplast DNA; Twinkle: T7 gp4-like protein with intramitochondrial nucleoid localization.

## Competing interest

The authors declare that they have no competing interests.

## Authors’ contributions

JDA performed the tissue-specific western blot and phylogenetic analyses and helped write the manuscript. BL helped conceive the project and made the recombinant protein construct and purified the protein, and performed some of the preliminary assays. JDC performed the qRT-PCR and western blot time course analyses. TH performed the DNA and amino acid sequence analyses. BLN helped conceive the project, performed the primase and helicase assays, and wrote the manuscript with JDA. All authors read and approved the manuscript.
